# Intelligent Online Monitoring of Rolling Bearing: Diagnosis and Prognosis

**DOI:** 10.3390/e23070791

**Published:** 2021-06-22

**Authors:** Hassane Hotait, Xavier Chiementin, Lanto Rasolofondraibe

**Affiliations:** 1 ITheMM, Institute of Thermics, Mechanics and Material, University of Reims Champagne-Ardenne, Moulin de la Housse, 51687 Reims, France; xavier.chiementin@univ-reims.fr; 2 CReSTIC, University of Reims Champagne-Ardenne, Moulin de la Housse, 51687 Reims, France; lanto.rasolofondraibe@univ-reims.fr

**Keywords:** bearings, vibration signal, DBSCAN, health indicator, LSTM, RUL

## Abstract

This paper suggests a new method to predict the Remaining Useful Life (RUL) of rolling bearings based on Long Short Term Memory (LSTM), in order to obtain the degradation condition of the rolling bearings and realize the predictive maintenance. The approach is divided into three parts: the first part is the clustering to detect the damage state by the density-based spatial clustering of applications with noise. The second one is the health indicator construction which could give a better reflection of the bearing degradation tendency and is selected as the input for the prediction model. In the third part of the RUL prediction, the LSTM approach is employed to improve the accuracy of the prediction. The rationale of this work is to combine the two methods—the density-based spatial clustering of applications with noise and LSTM—to identify the abnormal state in rolling bearings, then estimate the RUL. The suggested method is confirmed by experimental data of bearing life cycle, and the RUL prediction results of the model LSTM are compared with the nonlinear au-regressive model with exogenous input model. In addition, the constructed health indicator is compared with the spectral kurtosis feature. The results demonstrated that the suggested method is more appropriate than the nonlinear au-regressive model with exogenous input model for the prediction of bearing RUL.

## 1. Introduction

Rolling bearings, which is widely used in rotating machinery, are among the main parts of mechanical equipment. As the equipment runs, their performance will deteriorate and lead to failure. Consequently, early fault diagnosis and prediction of bearing life and safety are of high importance for predictive maintenance and the industrial reliability of mechanical equipment [1].

The vibration signals which are generated by rotating machines and collected can reflect the different processing conditions of modern industrial equipment. Industrial equipment is becoming more and more sophisticated and sensitive as technology evolves. For this reason, availability, reliability, and reduction of downtime and repairs are important. In that matter, condition monitoring has been determined as an effective means of increasing safety, health, and optimal equipment performance. Condition monitoring means the damage assessment and maintenance of machines without disturbing their operation and is performed based on information obtained from the condition of the equipment [2]. The vibration signals are used to provide real-time monitoring of the different states of bearings. The presence of intense vibrations in the rotating machinery usually implies the occurrence of faults in bearings. The vibration method is the base to start the data classification, and then detect the abnormal state of rolling bearings [3]. The density-based spatial clustering of applications with noise (DBSCAN) is an efficient method recently used to classify data [4]. The DBSCAN algorithm uses the signal density to separate the dense region from the noise region. Several analyses of bearing fault diagnostics and condition monitoring have been done as well as studying the prognostics of the defect in rolling bearings [5]. These predictions can reduce machine breakdowns, thereby minimizing the costs and support maintenance scheduling thus prolonging the life of the equipment.

Generally, data-driven prognostic frameworks comprise four stages: data acquisition, health indicator (HI) construction, degradation modeling and Remaining Useful Life (RUL) prediction [6]. Spectral kurtosis is used as a health indicator for wind turbine high-speed shaft bearings health prognosis analysis [7] as well as for the damage level assessment in bearing diagnosis [8]. Concerning the data acquisition, the diversity and the amount of sensor data collected in real-time must be enough to completely characterize the degradation performance of each component. For rotating machinery components, vibration signals are collected regularly, and time domain and frequency domain analyses are used for HI extraction [9]. One of the main queries in the prognostic of bearings is how to construct and evaluate health indicators from accessible features, which can represent the degradation states. The health indicator can be extracted from the time domain, frequency domain and scale domain [10].

For construction, the health indicator must reduce the number of features by using the Kernel Principal Component Analysis (KPCA) method [11] which is one of the most importantly used techniques. After the extraction of the health indicator, the mission is to choose the best model to predict the remaining useful health of the model. With the elaboration of sensor technologies, many data-driven methods along with statistical models have been developed to define product failures [12]. Particularly, the remaining useful life (RUL) of rotating machinery is described as the period between the present monitoring time and the failure time when the degradation signal surpasses a failure threshold level [13]. RUL prediction based on degradation signals has evolved as a crucial technology that gives failure information for health management and condition-based maintenance [14]. The data-driven RUL prediction methods that are widely used comprise regression models, Bayesian reasoning [15], Gaussian mixture models [16], and other statistical analysis methods, in addition to artificial intelligence methods such as fuzzy decision tree [17], artificial neural network (ANN) [18], and hidden Markov model [19]. The deep learning method is a hopeful tool to reach real-time bearing fault diagnosis, because it can provide a stable hierarchical feature representation from the raw bearing vibration signal and then investigate the bearing status online according to the real-time collected vibration signal. The importance of deep learning consists of its high potential to bypass false features caused by ambient noise and fluctuations in working conditions [20].

The purpose of RUL prediction is to obtain the deterioration trend of bearings at the present moment. In the part of RUL prediction, some samples are inserted into a Long Short Term Memory (LSTM) network in clusters as training sets to achieve model training and network parameter arrangement. The constructed model is evaluated with test sets, and the RUL prediction rates of these sets are attained. ANNs with various configurations has been utilized for short-term wind forecasting, where estimation beyond the last observations collected in the training data are only devoted to a few time steps [21]. An ANN (consisting of the input layer, hidden layer and output layer) is named shallow if it uses one hidden layer and deep if it utilizes more than one [22]. The recurrent neural network (RNN), is an inner structure of deep neural networks, as it makes the repair of complicated nonlinearities in the data feasible. It contains memory blocks with the possibility for reminding the information at each time from the preceding samples [23]. The LSTM is one of the well-known kinds of RNN for processing time series that can hold information for a long duration during the learning process [24]. Billings developed the Nonlinear Auto-Regressive with Exogenous Inputs (NARX) model as a new description of a large class of discrete, nonlinear systems [25]. Many systems have been examined by utilizing the NARX model [26]. It is another kind of RNN in which prediction of wind speed can be achieved by a procedure with additional meteorological time series data like air temperature and wind orientation [27]. Also, the NARX model can be used in other industrial structures such as modeling the major horizontal axis of wind turbines [28].

This study tests the predictability of rolling bearings by utilizing two different deep learning data-driven methods, the LSTM network model and the NARX that is also employed to compare and prove the effectiveness of the proposed method.

The achievability of the proposed process has been validated with experimental data sets. The following is a compendium of the major achievements of this paper:

(1) Data classification to detect an abnormal state in rolling bearings.
(2) Building of a special HI based on KPCA dimension reduction. This can efficaciously illustrate the degradation of rolling bearing operation.
(3) Presentation of a deep prognosis network—LSTM—which ameliorates the accuracy of the HI and RUL estimations significantly.
(4) Proof of the potential of the proposed methodologies based on experimental rolling bearing datasets. The results show that LSTM accomplished better execution than the NARX prognosis approach.

This paper is organized as follows: Section 1 is an introduction presenting a general description of the classification and reduction of dimensions to construct the health indicators, and finally the use of the LSTM neural network model and RUL. Section 2 describes the DBSCAN method used to classify and detect the degradation states. Section 3 presents the LSTM method to predict the remaining useful life of the studied equipment. Section 4 discusses the methodology proposed to detect the degradation state and predict the RUL. Section 5 presents the experimental verification of the suggested method. Then, Section 6 presents a comparison of the proposed methodology with NRAX. Lastly, Section 7 puts forth the conclusions of the paper.

## 2. DBSCAN Method

Density-Based Spatial Clustering of Applications with Noise (DBSCAN) is a well-known density-based clustering algorithm, first suggested by Ester [29], that is explained as a density-based clustering non-parametric algorithm in-favor-of a set of points in space. It is based on linking points that are closely stuffed together in high-density zones, thereby classifying as outliers the points that are isolated in low-density regions. It is considered one of the representative clustering algorithms and remarkably widely cited in the scientific literature. It implies the number of clusters based on the data rather than using it as requirement parameter. It can also detect clusters of arbitrary shape. The ε- neighborhood is essential for DBSCAN to estimate local densities, so the algorithm has two parameters: ε, which is the radius of a neighborhood near a data point p, in addition to MinPts, that is the minimal number of data points in a neighborhood needed to reveal a cluster.

Utilizing these two parameters, DBSCAN layers the data points in three categories:

*Core Points* are the foundations for clusters based on the density. The identical ε used to calculate the neighborhood for every point so the core points are data points that suited a minimal density requirement. A data point p is a core point if neighborhood (p, ε) [ε- neighborhood of p] includes at least MinPts; | neighborhood (p, ε) | ≥ MinPts.

*Border Points* are the points in clusters other than core points. A data point q is a border point if neighborhood (q, ε) comprises less than MinPts data points, however q is density-reachable of several core point p.

*Outliers* are points that are not referred to any cluster and maybe considered noisy points. The data point o is considered as outlier if it is none of the two, core point or border point. In other words, it is the “other” class.

The DBSCAN algorithm pseudocode can be expressed as the Algorithm 1:
**Algorithm 1.** DBSCAN algorithm pseudocode.Entries: DBSCAN (M, ε, MinPts)Outputs: Class setsInitialization arbitrarily choose a point pAs long as (there are still untreated points)Find all density-reachable points of p using Eps and MinPtsIf (p is a point of type center) a cluster is formedIf notIf (p is border type point and no point is reachable by density of p), the point p is considered as a noise and DBSCAN goes to the next point in the database.End

## 3. Long Short-Term Memory

Long short-term memory (LSTM) is a type of artificial recurrent neural network (RNN) [30] used in the field of deep learning. LSTM networks are used for classifying, processing, and making predictions based on time series data in the context of the occurrence of unknown duration delay between important events in a time series.

LSTMs were developed to treat the vanishing gradient issues that can be encountered when training traditional RNNs. They are superior to RNNs, hidden Markov models, and other sequence learning methods in several applications [31]. The advantage consists predominantly in the LSTMs’ ability to hold long-term memory, whereas due to the short-term memory, the typical RNN will only be capable of utilizing the contextual information from the last data which is not helpful at all [32]. They also have a multifactorial structure. At every time step, the LSTM cell considers three different factors: the current input data, the short-term memory (hidden state), and lastly the long-term memory (cell state). The cell then uses gates to analyze the information to be kept at each time step before reaching the long-term and short-term information to the next cell. These gates are called the Input Gate, the Forget Gate, and the Output Gate [33]. These gates are defined as follows:

*Input Gate.* This chooses what new information will be kept in the long-term memory. It only handles the information from the present input and the short-term memory from the preceding time step, so it has to extract the non-useful information from these variables.

*Forget Gate***.** It settles which information from the long-term memory should be preserved. This is achieved by multiplying the incoming long-term memory by a forget vector produced by the present input and incoming short-term memory.

*Output Gate.* This will use the present input, the preceding short-term memory, and the recently computed long-term memory to obtain the new short-term memory, which will be moved on to the cell in the following time step. The output of the present time step can also be displayed from the hidden state.

**Figure 1:**
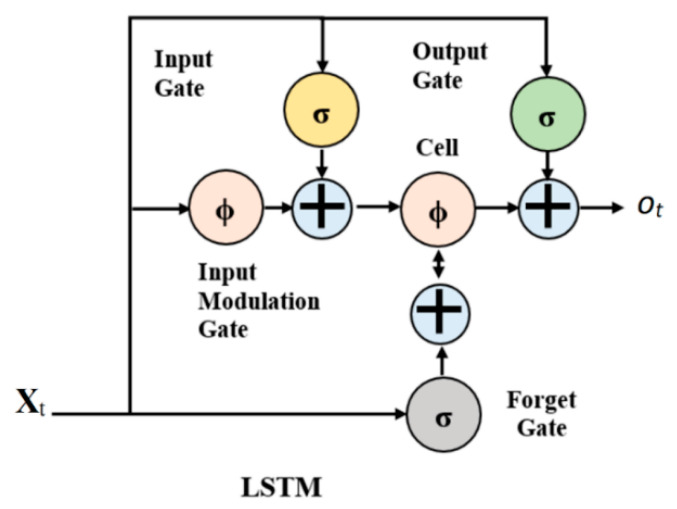
The LSTM module, where Xt,$\;i_{t}$, $o_{t}$ are input, and output gates, respectively.

A typical LSTM module, also known as a repeating module, has four neural network layers linked to each other uniquely as shown in Figure 1. The module has three-gate activation functions σ and two output activation functions ϕ as described in Figure 1. This is usually similar to replacing the addition operation (+) by a Hadamard product (ʘ) in the standard Elman-RNN equation. The concatenation operation is represented by the symbol (ʘ) bullet. The network can indicate the amount of previous information to flow. It is controlled through the first layer (σ), given by Equation (1) [34].

Given that Xt the input gate activator vector at time t, it the forget gate activator vector at time t, ot the output gate activator vector at time t, ct the outcome of cell vector at time t, ht the outcome of layer vector at time t, Wx, Wi, and Wo the input kernels for the respective gates, Uf, Ui, and Uo, the recurrent kernels for the respective gates, *b_f_*, *b_i_*, *b_o_*, and *b*_c_ the biases, σ the logistic sigmoid function, ʘ the matrix product, and *tanh* the hyperbolic tangent activation function.

*x_t_* = σ(**W**_x_x_t_ + **U**_f_h_t_−_1_+ *b_f_*) (1)

*i_t_* = σ(**W**_i_xt + **U**_i_h_t_−_1_+ *b_i_*) (2)

*o_t_* = σ(**W**_o_x_t_ + **U**_o_h_t_−_1_+ *b_o_*) (3)

*c_t_* = f_t_ ʘ c_t_−1+ i_t_ ʘ *tanh* (**W**_c_x_t_ + U_c_h_t_−_1_+ *b_c_*) (4)

*h_t_* = o_t_ ʘ *tanh* (c_t_) (5)

The current information to be stored in the cell state is generated using two network layers. A sigmoid layer (σ) that establishes values to update ($i_{t}$) (Equation (2)) and tanh layer ϕ that details a vector of new possible values ($o_{t}$) as shown in Equation (3). The fusion of both is then added in the state. Lastly, the cell state is modified using Equation (5) [35].

## 4. Details of the Proposed Methodology

**Figure 2:**
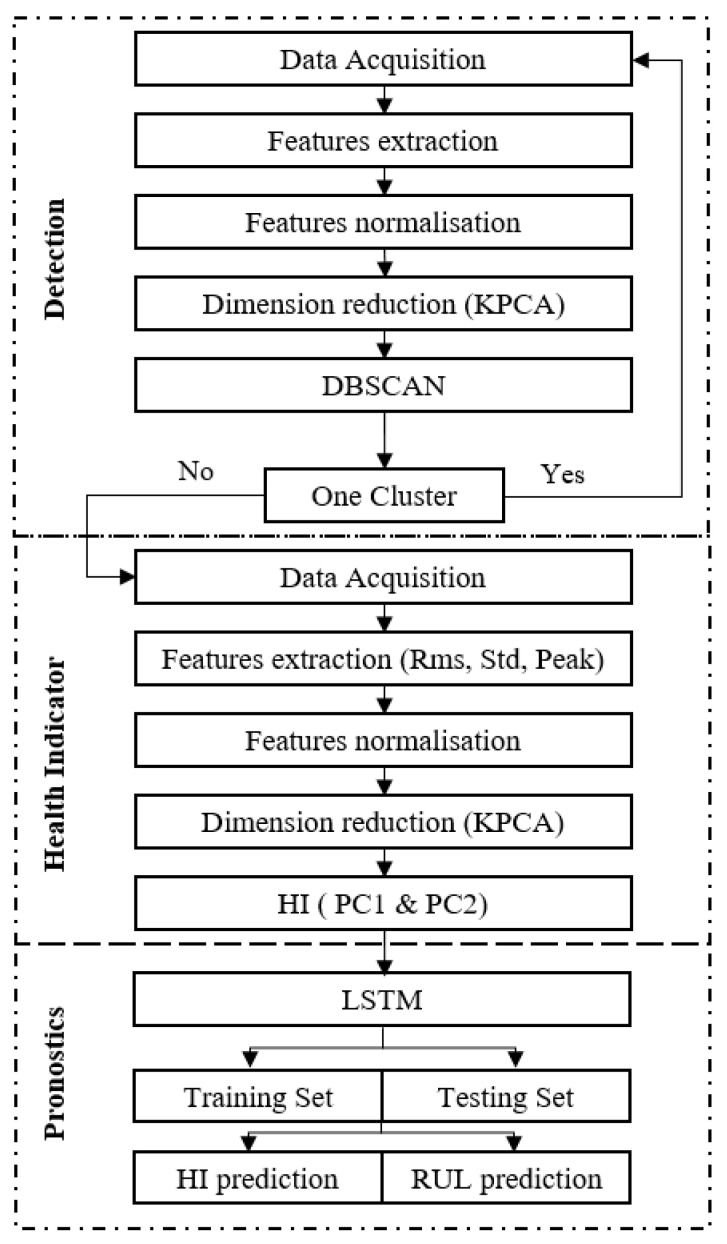
Flowchart of the proposed methodology.

**Figure 3:**
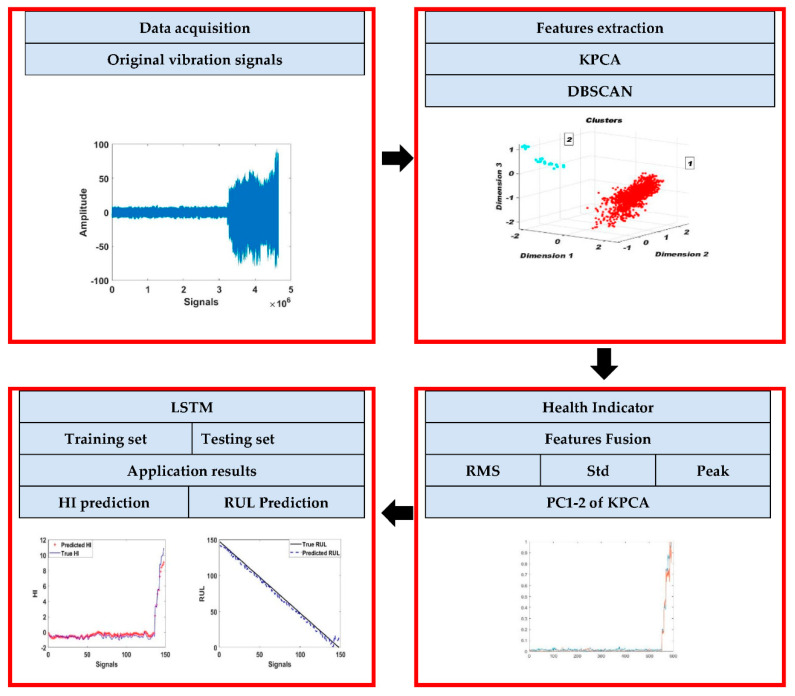
The procedure of the proposed methodology.

The proposed methodology consists of online-integrated chains that are shown in Figure 2 and Figure 3, which make it robust. It starts by determining the healthy state of rolling bearings and then stratifying the data into healthy and faulty. After detection of the defect, the prognostics neural network is constructed, that aims to calculate the remaining useful life. The objective of this methodology is to detect the defect by the DBSCAN method, then calculate the remaining useful life by the LSTM method to predict the equipment degradation state evolution.

Figure 1 reflects the proposed approach, which is divided into three parts: the first part is the clustering to detect the damage state, the second one is the health indicator construction, the third part of the RUL and HI prediction.

### 4.1. The Proposed Framework

The chain begins with the data acquisition by collecting signals, then extracting features. The collected features are described in Section 4.2 Feature Extraction Methods, where they are separated into a time-domain. After their extraction, these features are normalized by the z-score method [36] which is described by Equation (6). The KPCA method was used to reduce the number of features and show them in 3D. The DBSCAN parameters Minpts and Epsilon are defined in this stage. The calculation of the Minpts value depends on the initial matrix T which is equal to the first two successive moments (every successive moments contain eight signals) and the Minpts value is equal to ($\frac{1}{2}T)$.

The epsilon is calculated by the nearest neighbor method for the initial matrix (Equation (7)). The estimation of epsilon value is realized by generating a k-distance graph for the input data MF. For every point in MF, the distance to the k-th nearest point is calculated, then arranged points at this distance are plotted. The graph includes a knee. The distance connected to the knee is mostly a good choice for epsilon since it is the region where points start appending off into outlier (noise) region [37].

Prior to plotting the k-distance graph, the Minpts that ae the short pairwise distances for examinations in MF have to be sited in ascending order. The features matrix MF, is normalized ${\left[MF\right]}_{i}^{norm}$ (Equation (6)). The objective of normalization is to transform the calculated values to be on a similar scale:

(6) $${\left[MF\right]}_{i}^{norm}=\frac{MF_{i}-\bar{MF_{i}}}{std\left(MF_{i}\right)}$$

ε corresponds to the maximum distance between the center of the class, $c_{h}$ and the ${\mathrm{MinPts}}^{\mathrm{th}\;}$neighbor. The outcoming class is so-called healthy class, well known $C_{h},$ with center $c_{h}$. This class represents a reference state:

(7) $$\varepsilon =\mathrm{distance}\left(c_{h},{\;\mathrm{MinPts}}^{\mathrm{th}\;}\mathrm{neighbor}\right)$$

The DBSCAN objective is to detect the degradation state. When two or more classes are detected the first one is the normal state, the second and the third are the degraded ones. This chain returns to the acquisition phase under the condition of not detecting the second class. This classification ends with the detection of the second class, which is the degraded class [38].

The prognostic’s objective is to show the evolution of the degraded state of the equipment and calculate the RUL. The health indicator is divided into trained and tested data [39]. After that, the application of the regression model will be done by the LSTM method to predict the remaining useful life.

### 4.2. Health Indicator

In the field of fault diagnosis, the time domain features are an essential index to detect the health condition of mechanical equipment, three pertinent time domain features are combined and fused with the KPCA method to construct the health indicator, which can be defined as follows in Table 1.

**Table 1:** Health indicator features.

Root mean square:$\;\;RMS={\left(\frac{1}{N}\overset{N}{\underset{i=1}{\sum }}\left(x_{i}^{2}\right)\right)}^{\frac{1}{2}}$	(8)
Standard deviation: $\left(Std\right)={\left(\frac{1}{N}\overset{N}{\underset{i=1}{\sum }}{\left(x_{i}-\bar{x}\right)}^{2}\right)}^{\frac{1}{2}}$	(9)
Peak to Peak: $x_{PEAK}=max\;\left(x_{i}\right)-min\;(x_{i})$	(10)

HI construction algorithm (Algorithm 2).
**Algorithm 2.** HI construction algorithm.**Input**: raw life-cycle data; combine (RMS, Std, Peak) features; Sensitive health features selection. 1. Construct a feature space with 3 features referred to in Table 1 by time-domain, from raw life-cycle data. 2. Reduction of the dimension of 3 features by KPCA. 3. Minimize the weights of each input-sensitive health feature by KPCA. 4. The first Two principal component (1–2) represents the maximum variance direction in the data. 5. Find the sensitive health features by the predetermined termination. 6. Construct the optimal one-dimensional HI. **Output**: HI; sensitive health features.

### 4.3. Feature Extraction Methods

The multi-domain feature set can completely represent the bearing fault feature state which can provide an efficient diagnosis for different rolling bearing faults occurring under varying speed and load or anonymous speed conditions. The existence of defects inmachinery components can be observed in the raw acceleration signals. To get a better comprehension of the vibration signals we attempt to extract the time domain, frequency domain, and time-frequency domain features [36].

Time-domain features of vibration signals have proved to be useful to present the machinery condition. The time-domain features were used to detect bearing damages such as kurtosis, standard deviation, skewness, root mean square efficiency for detection, and localized bearing default. In addition to the peak-to-peak value, impulse factor, Tikhat, Talaf, crest factor, that can detect the changes in the signal when a defect occurs. These classical time-domain statistical features were applied to represent the bearing condition [40].

The frequency-domain is based on the spectral analysis technique that can convert time-domain vibration signals into discrete frequency components using a fast Fourier transform (FFT) [41]. The signal spectrum contains rich condition information. The frequency root mean square$\;f_{rms}$, and the frequency root mean square brut:$f_{rmsb}$_,_ the frequency center:$\;f_{c}$, standard deviation frequency brut:$\;f_{stdb}$, the power envelope: PW, the mean frequency $f_{rmsf}$ are used in the rolling bearing to detect the defect [42].

The time-scale analysis methods decompose the extracted signals into a set of scale components that contain several fault features. The wavelet process has an advantage over the conventional Fourier transform in the condition of the analysis of discontinuous and short-impulse signals [43]. Wavelet is a well-known signal processing technique used to examine the frequency composition of the signal [44]. Therefore, technical description will be omitted. Instead, the two new features extracted will be introduced: WRMS, which is the effective value of the frequencies of the signal’s wavelets spectrum, and PCWT that represents the average value of the envelope amplitudes [45].

### 4.4. Reduction of Dimensions

The reduction of dimensions is a step used to reduce the number of features and easily plot the observed data in 3D or 2D. There is often a correlation between the feature dimensions and the fault detection. Thereby, it is very significant to extract the critical fault features that reproduce the fault and reduce the dimension data. KPCA is a simple dimension reduction method which is an extension of PCA. It is an efficient tool for multivariable and nonlinear data, and its major functions are that it plots the indigenous spatial data into high dimensional space by kernel function, converts the original nonlinear issue to linearization one, and utilizes PCA to minimize the dimensions [44].

### 4.5. Evaluation of Remaining Useful Life (RUL)

The performance of the RUL estimation procedure has been tested extensively to evaluate the RUL in the fitted LSTM method. According to the criteria adopted in the methodology (Figure 2), three evaluation criteria are taken into consideration to measure the prediction performance of different approaches:

#### 4.5.1. Root Mean Square Error (RMSE)

Root mean square error is often used to measure of the differences within predicted values by a model or observed and estimator values. The RMSE represents the square root of the second sampling time of the differences between predicted and observed values or the root mean square of these differences [46].

The RMSE of prognosticate values $y_{i}\;$for times t of a regression’s count on variable $x_{i}\;$with observed variables over n are the signals number. It is calculated for T different predictions and is equal to the square root of the mean of the squares of the deviations:

(11) $$\mathrm{RMSE}=\sqrt{\frac{\sum_{i=1}^{n}{\left(y_{i}-x_{i}\right)}^{2}}{T}}$$

#### 4.5.2. Mean Absolute Error (MAE)

Mean absolute error (MAE) is the calculation of errors among paired observations noticing the same occurrence. Patterns of Y versus X include contrasts of predicted against observed, initial time versus subsequent time, and unitechnique versus an alternative technique of measurement [47]. MAE is calculated as:

(12) $$\mathrm{MAE}=\frac{\sum_{i=1}^{n}\left|y_{i}-x_{i}\right|}{n}=\frac{\;\sum_{i=1}^{n}\left|e_{i}\right|}{n}$$

The mean absolute error is calculated as follows: $\left|e_{i}\right|=\left|y_{i}-x_{i}\right|$ where$\;y_{i}\;$is the prediction value and $x_{i}\;$is the true value. It utilizes the same scale as the existent in the measured data and is a familiar measure of forecast errors in time series analysis.

## 5. Experimental Validation

### 5.1. Test Bench

The tests were performed on a group of identical single-row thrust bearings. The objective was to follow the development of spalling from micro to fatigue state. These tests were performed on a fatigue module of a test bench. A constant axial load of 3000 daN, was applied to the bearing with the rotation speed remained constant during the tests, i.e., 1800 rpm. The coolant flow was also constant. Two type DJB3208 and DJB3209 piezoelectric accelerometers were placed as close as possible to the bearing in two different directions, axial and radial. The used data is the data of the piezoelectric sensor positioned radially on the bearing is taken as the best measuring point. The OROS OR34 acquisition system recorded 8192 points of the vibratory signal in a frequency range of 20 kHz to have a significant number of cycles.

**Figure 4:**
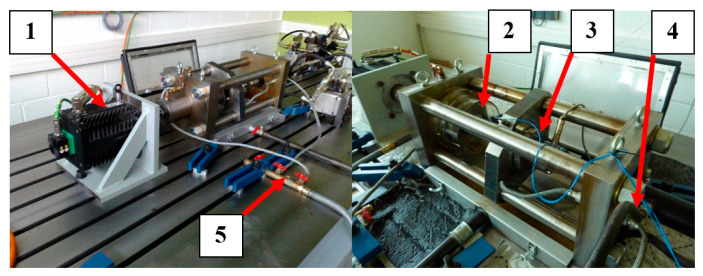
Module of the thrust bearing fatigue test bench.

The test bench shown in Figure 4, is essentially composed of an electric motor (1), that turns a spindle (2) on which one of the bearing rings is mounted. A shaft (3) transmits the axial load to the thrust bearing from a hydraulic pump (4), and a continuously operating lubrication circuit (5) for cooling. This test bench is connected to a control and data acquisition system. The ball bearings used are of type FAG 51207 CZECH/ATK. This bearing allows easy disassembly and immediate visual inspection of defects.

### 5.2. Procedure of the Experiments

**Figure 5:**
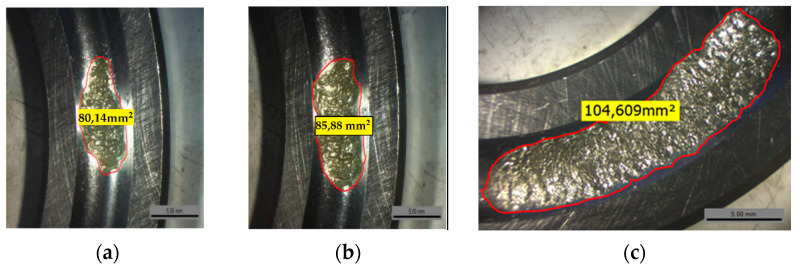
The spalling bearing of the three tested rolling bearing for the bearing number 1 (**a**,**b**) for bearing number 2 and (**c**) for the bearing number 3.

During the tests, the temperatures of the main shaft bearing, the lubricating oil, and the ambient air were continuously monitored. As shown in Table 2, three bearings were used and subjected to a fatigue phenomenon until the bearing broke, Figure 5. The health indicator was built from the first two principal components which covered 99.1% of the original data but the first component represented less with 97.9%.

The cases 1–3 were applied on the proposed method LSTM to get the predicted health indicator and RUL. Knowing that cases 1 and 2 were the training cases of the bearing 1 with the testing of the bearings 2 and 3, respectively. As well, case 3 was the training of the bearing 2 with the testing of the bearing 3, with 25% of the training data being tested in each case.

### 5.3. Results of the Experiments

**Figure 6:**
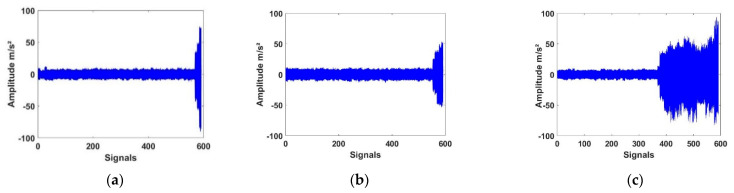
The time-domain waveform of bearing, (**a**) number 1, (**b**) number 2, and (**c**) number 3.

Three different conditions were considered throughout the experiment, operating with different spalling sizes: the first one with 80.14 mm^2^, the second one with 85.88 mm^2^ and the third one with 104.6 mm^2^. The measured vibration signals of bearing numbers 1–3 are exhibited in Figure 6a–c. During the tests, every bearing was naturally degraded. As a result, every test bearing collected a different vibration signal pattern.

As shown in Figure 4, the entire lifetime vibration signals of three test bearings were demonstrated. The signal amplitudes of bearing 1 displayed a fast increase near the end of life which designated a sudden degradation. The signal amplitudes of bearings 2 and 3 had a trend of progressive increase. This showed that degradation began to change slightly at first and then in a severe way.

**Figure 7:**
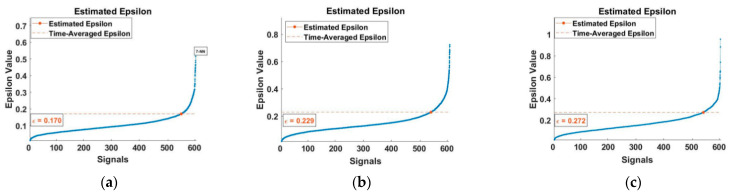
Estimated epsilon, (**a**) for bearing number 1, (**b**) for bearing number 2, and (**c**) for bearing number 3.

To determine the unique optimal value Eps, rate determination for single-level density was obtained by calculating the slope between points. Figure 7 depicts the result of a plot that has been sorted in ascending values. Detection of Eps value was done by calculating the slope of the lines of four Minpts values. The slopes of the lines were located for the three bearings at the points of 0.170, 0.229 and 0.272, which are the optimal values Eps for bearings 1, 2 and 3, respectively (Figure 7a–c).

**Figure 8:**
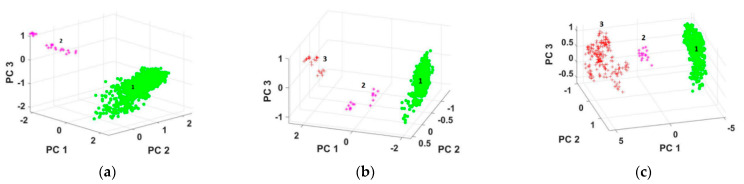
DBSCAN classification, (**a**) for case number 1, (**b**) for case 2, and (**c**) for case number 3.

The DBSCAN method to classify bearing fault was constructed and tested to predict the conditions of the bearings, then the performance of the classification was evaluated. The results are shown in Figure 8 for the three bearing numbers. Figure 8a, represents two classes: the first one being the healthy state with 568 signals, and the second one illustrating the first stage of the defect, starting from signal number 569. Figure 8b, displays three classes, the first class being the healthy one with 560 signals, the second one representing the first stage of the defect, starting with the signal number 561, and third class continuing from signals number 577 to the end and representing a severe defect. Figure 8c illustrates three classes, the first one showing the healthy state with 480 signals, the second class goes from the first stage of the defect, starting with the signal number 481, and the third class starting from signals number 516 to the end represents a severe defect.

The results were proportionally correlated to the amplitude, size of defect, value of epsilon, and the detected classes. The size of defect increased with the rise of the Eps value resulting in an augmentation of the classes number. The performance of the proposed method for calculating the bearing health indicator is based on the fusion of the three features, standard deviation, RMS, and peak, which are popular choices for bearing health indicators, then extracting from them the first two principal components.

**Figure 9:**
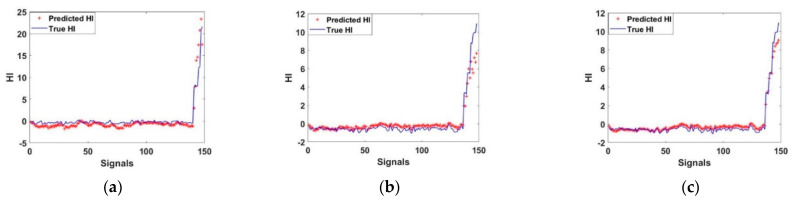
LSTM Regression, (**a**) case 1, (**b**) case 2, (**c**) case 3.

In Figure 9, the predicted HI and true HI of rolling bearing are demonstrated. As a result, LSTM regressions for the predicted HI and true HI for testing cases 1–3 are shown in Figure 9a–c, respectively. In the third case, the LSTM regression of the predicted HI and true HI were close to each other which indicated that this case was better than cases 1 and 2.

**Figure 10:**
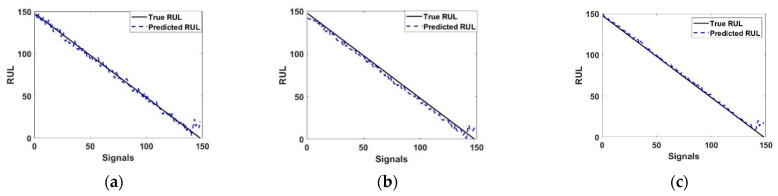
RUL prediction of LSTM in (**a**) case 1, (**b**) case 2, (**c**) case 3.

Figure 10 shows the results of the predicted RUL and the true RUL value, where the error between the predicted and true was minimal. To achieve RUL prediction, suitable reliability indicators must be established. Based on the current mapping feature set, the reliability assessment model was firstly utilized to evaluate the precision of the mapping time domain. Then, to build the mathematical exemplification relationship among the component degradation procedure parameters and the prediction RUL model, an LSTM algorithm was utilized in this section. It was established that the accuracy indicator attenuation path was determined prior to the current life and modified to be a fraction of prediction after the present life [47].

Case 3 represented in Figure 10c confirms the results shown in Figure 9c, meaning that predicted and true RUL were linear. Moreover, this case represented bearing numbers 2 and 3 which are the best to predict RUL because they have the same degradation state with the three classes of defect.

## 6. Discussion

**Figure 11:**
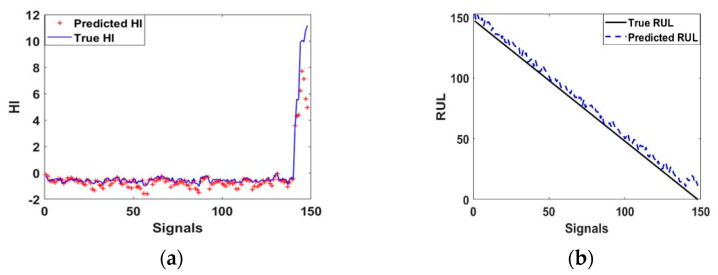
(**a**) HI Regression, (**b**) RUL prediction.

To avoid the volatility caused by the trend prediction of the curve by the LSTM method, the value was taken as the evaluation standard, as shown in Table 3. The next phase comprised validation of the trained and tested models. The NARX model’s results shown in Figure 11a,b, present respectively the final results of the model obtained from case 3. Then, the comparison of the performance was done between the LSTM model and the NARX one. The mean absolute error (MAE), the root mean square error (RMSE), and accuracy were adopted to evaluate the performance of the two different prediction models.

As shown in Table 3, the LSTM method case 3 with the proposed HI has the minimum MAE value compared with NARX, and the average MAE value for the LSTM method on datasets of different percentages was 0.007. The NARX method has the biggest MAE with an average value of 0.03. Compared with MAE, the RMSE of the prediction models was larger. For the LSTM method, the average RMSE on different testing datasets was 0.04. Moreover, this metric for the NARX method even reached 0.4. The accuracy value for the LSTM method on datasets of different percentages was 93%. The NARX method has the smallest accuracy with the kurtosis spectral like a health indicator with an average value of 86%. These values showed that the effectiveness of the LSTM method remaining useful life prediction is promising. The elapsed time is the time required by the algorithm to give a response. The LSTM method gave a faster response compared with NARX method.

## 7. Conclusions

This paper proposed the LSTM method to extract high-quality degradation models and predict remaining useful life of rolling bearings from their vibration signals. The extracted bearing vibration signal features included nine time-domain features that were used in the detection chain. The health indicator was constructed from the fusion of three-time domain features: RMS, Std and Peak.

The results showed that the suggested method can adjust to varying operating conditions. The LSTM method was adopted for bearing degradation state and prediction of the remaining useful life. The performance of the proposed method on the dataset was compared with the NARX method whom accuracy is 90%. The results present the superiority and effectiveness of the proposed methodology from the RMSE, MAE and their accuracy are 0.04, 0.007 and 93%, respectively.

The objective of bearing fault diagnosis is to consolidate an efficient real-time condition monitoring and recognition system to make continuous production over predictive maintenance. In order to realize this goal, there are two main topics for future work:

(1) The fault diagnosis model requires the performance of continual and rapid diagnosis situated on the vibration signals extracted in real-time.
(2) The good performance was maintained under various working conditions, and the application of the fault diagnosis model must be generalized under different load and noise conditions.

As future work, we plan to examine the use of other algorithms for resolving bearing remaining useful life prediction issues and set up cooperative predictions under different working conditions.

## Figures and Tables

**Table 2:** Summary of fatigue tests on bearings.

Bearing Number	Signal Number	Spalling Size (mm^2^)
1	592	80.14
2	592	85.88
3	592	104.6

**Table 3:** Evaluation of predicted useful life.

	Data	Methods	LSTM	NARX
Proposed HI	Experimental Case 1	RMSE	0.05	0.3
		MAE	0.01	0.02
		Accuracy %	89	87
		Time Elapsed	49 s	52 s
	Experimental Case 2	RMSE	0.3	0.6
		MAE	0.01	0.02
		Accuracy %	85	81
		Time Elapsed	49 s	52 s
	Experimental Case 3	RMSE	0.04	0.4
		MAE	0.007	0.03
		Accuracy %	93	90
		Time Elapsed	49 s	52 s
Kurtosis Spectral	Experimental Case 3	RMSE	0.1	0.4
		MAE	0.02	0.03
		Accuracy %	86	85
		Time Elapsed	49 s	52 s
